# Diatoms and pollen data from modern surface sediment samples collected from the Merang wetlands, Kuala Terengganu, Malaysia

**DOI:** 10.1016/j.dib.2018.10.156

**Published:** 2018-11-03

**Authors:** Cheuk-Yan Tam, Yongqiang Zong, Haixian Xiong, Zhuo Zheng

**Affiliations:** a Department of Earth Sciences, The University of Hong Kong, Hong Kong SAR, China; b School of Earth Science and Geological Engineering, Sun Yat-sen University, Guangzhou, China

## Abstract

The data presented here are related to the research paper entitled “A below-the-present late Holocene relative sea level and the glacial isostatic adjustment during the Holocene in the Malay Peninsula” (Tam et al., 2018) [1]. The diatoms and pollen data are collected from surface sediments of the Merang wetlands, Kuala Terengganu, Malaysia, and are presented as percentages of total diatoms or total land pollen respectively. Ground elevations of the sampling sites are levelled to the national datum and expressed as elevations above or below mean sea level. These diatom and pollen data can be used for indicative meaning calibration of sea-level index points and for the development of diatom-based or pollen-based tidal level transfer functions. These data have been used for calibrating the indicative meanings for sea-level index points in the reconstruction of Holocene sea-level history of the Peninsular Malaysia.

**Specifications table**

**Table:** 

Subject area	*Quaternary geological science*
More specific subject area	*Sea-level and coastal studies*
Type of data	*Table, figure*
How data were acquired	*Field survey, Russian corer, microscope, Tilia (software)*
Data format	*Percentages of total diatom or land pollen analyzed*
Experimental factors	*Diatom and pollen assemblages from modern surface sediment samples were used for establishing the microfossil-elevation relationship. This relationship will later be used to quantify the indicative meaning of a sea-level index point.*
Experimental features	*Sediment samples were collected at various elevations of land surface including tidal mudflat, mangrove and back mangrove forests, tidal inlets and channels and freshwater wetlands. Each sample was treated according to standard methods to extract microfossil diatom valves and pollen grains. Under a microscope, diatoms were identified to species level, with individuals counted. Pollen grains were identified to genus and/or family levels, with individuals counted.*
Data source location	*The Merang Wetlands, Kuala Terengganu, Malaysia*
Data accessibility	*Data are with this article*
Related research article	*Tam, C.-Y., Zong, Y., Kamaludin, b. H., Hamlee, b. I., Habibah, b. J., Xiong, H., et al., 2018. A below-the-present late Holocene relative sea level and the glacial isostatic adjustment during the Holocene in the Malay Peninsula. Quaternary Science Reviews 201, 206–222.*

**Value of the data**

• This is the first diatom and pollen data set from surface sediments collected from the Malay Peninsula since Kamaludin [2] and Zong and Kamaludin [3]. This data set can help establish the microfossil-elevation relationship in tropical mangrove environments. More specifically, this data set can help quantify the indicative meaning of a sea-level index point collected from such environments.
• As the elevations of the sampling sites are leveled to the national datum, this data set can be merged with any other similar data sets to form a larger data set that shows the elevational relationship between microfossil diatom/pollen and tidal water levels.
• This data set can assist the reconstruction of past sea-level history and coastal change in tropical regions.

## 1 Data

**Fig. 1:**
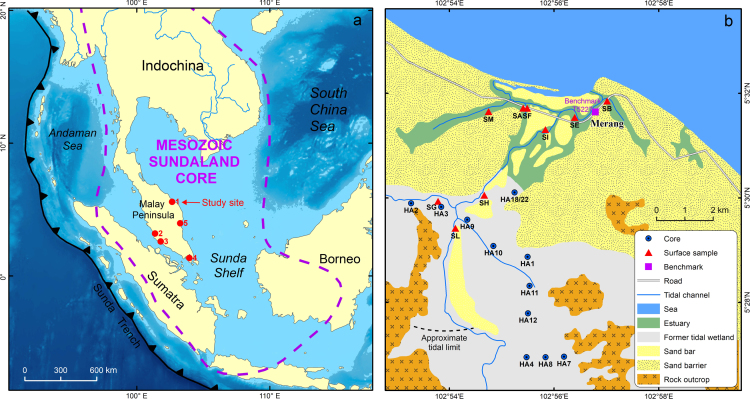
Map A illustrates the location of the site, from which the diatom and pollen data were obtained. This site is located on the east coast of the Peninsula Malaysia within the Mesozoic Sundaland Block, a tectonically relatively stable area. Map B illustrate the detailed landscape of the Merang wetland, near Kuala Terengganu, and specific locations where the surface sediment samples were collected.

The data set covers an elevational range between −0.20 and 1.30 m, i.e. between local mean low waters and mean high higher waters. Fig. 1a indicates the location of the study site. Fig. 1b shows the sampling locations where the surface sediments were collected. Table A presents the diatom data from each of the sampling point along with the ground elevation. Figure B presents the pollen data from each of the sampling point along with the ground elevation.

## 2 Experimental design, materials and methods

The data set was generated from a study site that lies about 25 km northwest of Kuala Terengganu of the Malay Peninsula (Fig. 1a). The landform of this site is a barrier–estuarine–lagoon system, called the Merang wetland (5°26′32″N; 102°52′58″E; Fig. 1b). Locally it has a microtidal regime and it is under a humid tropical climate. Details of the landscape can be found from several recent surveys [4], [5]. At present, the barrier on the seaward side of the wetland is about 4–5 km wide and 3–4 m above mean sea level (MSL), mostly covered by farm activity and the distinctive natural vegetation called locally as ‘Gelam’ [6]. This vegetation is characterized by a species of Myrtaceae (*Melaleuca cajuputi*) that tends to grow on mangrove peaty soils and can survive flooding of tidal water [7]. Between the barriers and an estuary, which is about 2 km wide, occupied by large sand bars and multiple tidal channels. Behind the barriers, a former lagoon is fully filled with estuarine and mangrove sediments.

Within the estuary, 43 modern surface sediment samples were collected from a number of locations (Fig. 1b). In each location, several natural representative habitat zones were identified. According to the habitat zones, several modern surface sediments were sampled in each location nearly evenly along an elevational gradient. The ground elevation of each sampling point was obtained by levelling from the sampling point to the local JUPEM (Department of Survey and Mapping) benchmark (Fig. 1b) using a Total Station surveying system. The JUPEM benchmark provides mean sea level elevational information referred to the Peninsula Malaysia vertical datum [4].

The collected modern sediment sub-samples were processed following the procedures of Faegri and Iversen [8] for extraction of pollen grains and preparation of slides. The procedures of Zong and Sawai [9] were adapted for extraction of diatoms and preparation of slides. Under a microscope, over 300 land pollen grains were counted normally for each sample except for a few samples that have very low pollen concentrations. References used for identification include Rao and Lee [10], Huang [11], Somboon [12], Wang [13], Kamaludin [2], Li et al. [14] and Mao et al. [15]. The online data from the Australasian Pollen and Spore Atlas [16] were also consulted. The percentages of each taxon are calculated based on total pollen sum. Similar to the pollen analysis, over 300 diatom valves were counted from each sample. The diatom taxa are grouped into the five categories: marine water, brackish water, freshwater salt tolerant, freshwater and freshwater salt intolerant [9], [17], [18]. Identification for diatoms was based on [19], [20], [21], [22].
