# Supplementary material for: Diatoms and pollen data from modern surface sediment samples collected from the Merang wetlands, Kuala Terengganu, Malaysia
Source: Data Brief. 2018 Nov 3;21:1886–9. doi: 10.1016/j.dib.2018.10.156 (PMC6260407; doi:10.1016/j.dib.2018.10.156)
Supplement: Supplementary file 2 — Supplementary material [file mmc2.docx]

Percentages of total diatoms from each modern surface sediment sample collected from the Merang wetlands, Kuala Terengganu, Malaysia

**Site SB1 SB2 SA1 SB3 SM13 SA2 SM11 SA3 SM12 SM09 SM03 SM14 SM15 SM16 SH2 SH3 SL1 SL2 SL3**

**Elevation (m, MSL) +0.95 +0.88 +0.60 +0.50 +0.40 +0.39 +0.38 +0.28 +0.24 +0.17 +0.14 -0.01 -0.09 -0.15 +0.09 -0.41 -1.19 -0.23 +0.19**

Achnanthes brevipes 0.24 1.23 0.63 1.81 1.96 3.19 1.55 1.64 3.23 1.35 6.90 6.09 4.61 2.43 0.00 0.00 0.00 0.00 0.00

Achnanthes delicatula 0.24 0.62 0.63 1.48 2.69 4.35 0.52 8.22 3.52 1.35 1.57 2.03 1.44 0.87 1.17 1.30 0.00 0.00 0.00

Actinoptychus senarius 0.00 0.00 0.00 0.00 0.00 0.00 0.52 0.33 0.59 1.08 0.94 1.45 1.15 1.04 0.00 0.00 0.00 0.00 0.00

Amphora coffeaeformis 14.59 12.96 20.66 32.24 41.32 25.51 19.59 15.13 25.51 39.35 21.32 25.51 33.14 28.82 6.82 11.48 1.99 2.54 2.37

Amphora ovalis 2.15 0.62 0.00 0.00 0.00 0.00 0.00 0.00 0.00 0.00 0.00 0.00 0.00 0.00 4.48 6.48 3.97 6.50 4.75

Aulacoseira islandica 0.00 0.00 0.00 0.00 0.00 0.00 1.03 0.66 0.88 1.35 0.31 0.58 1.15 0.52 1.56 3.33 11.92 11.86 23.22

Caloneis westii 0.48 0.62 0.00 0.00 0.00 0.00 0.00 0.00 0.00 0.00 0.00 0.00 0.00 0.00 0.00 0.00 0.00 0.00 0.00

Chaetoceros radians 0.00 0.00 0.00 0.00 0.00 0.00 0.77 0.66 0.29 0.54 1.25 1.45 1.73 2.08 1.17 0.93 0.99 2.26 0.79

Cocconeis placentula 0.00 0.00 0.00 0.00 0.00 0.00 0.00 0.00 0.00 0.00 0.00 0.00 0.00 0.00 4.48 4.07 6.95 8.19 3.43

Cocconeis scutellum 0.00 0.62 2.37 0.49 1.96 2.90 2.84 2.30 2.35 1.35 0.94 2.32 2.02 4.34 1.56 2.78 0.00 0.00 0.00

Cosinodiscus obscurus 1.44 1.23 2.84 0.82 1.22 4.06 4.12 6.25 3.52 2.16 1.25 2.03 1.44 1.39 2.34 4.81 0.00 0.28 0.26

Cyclotella striata 0.72 1.85 2.21 0.33 2.20 5.22 4.12 3.95 4.40 1.89 1.25 2.61 1.44 2.78 6.34 2.78 0.00 0.56 0.79

Cymatosira belgica 0.00 0.00 0.00 0.00 0.00 0.00 0.77 0.33 0.59 0.81 0.31 1.16 0.86 0.87 0.00 0.00 0.00 0.00 0.00

Cymbella turgida 0.00 0.00 0.00 1.64 5.38 2.61 5.15 1.64 2.35 3.23 3.76 2.61 2.31 3.12 0.97 3.33 1.99 3.67 2.64

Denticula tenuis 0.00 0.00 0.00 0.00 0.00 0.00 0.00 0.00 0.00 0.00 0.00 0.00 0.00 0.00 0.39 0.00 0.00 0.00 0.00

Diploneis elliptica 0.00 0.00 0.00 0.00 0.00 0.00 0.00 0.00 0.00 0.00 0.00 0.00 0.00 0.00 1.75 4.07 5.30 3.11 2.90

Diploneis interrupta 0.00 0.00 0.00 0.00 0.00 0.00 0.00 0.00 0.59 1.08 0.63 2.61 3.17 0.52 8.77 3.33 2.65 4.24 6.07

Diploneis smithii 0.00 0.00 0.95 1.81 1.96 1.74 5.93 2.96 4.11 5.12 4.70 4.06 3.75 6.42 2.92 1.85 0.00 0.28 0.79

Eunotia tenella 9.81 4.32 1.10 3.62 0.98 0.87 2.06 0.66 0.88 0.27 0.00 0.29 0.58 0.52 5.46 0.56 7.95 4.24 1.32

Fallacia subhamulata 42.11 54.94 2.37 0.33 1.22 0.00 0.77 0.00 0.00 0.54 0.00 0.00 0.00 0.69 3.12 1.48 0.99 1.69 2.11

Fragilaria pinnata 0.00 0.00 0.00 0.00 0.00 0.00 2.32 0.00 0.00 0.27 0.00 0.00 0.58 0.52 1.36 1.30 7.28 6.78 1.58

Frustulia rhomboids 1.67 3.70 0.63 0.00 1.71 0.00 1.55 0.00 0.00 0.00 0.00 0.00 0.29 0.00 7.21 9.44 12.58 7.91 3.69

Gomphonema parvulum 2.15 0.62 0.16 0.16 0.49 0.00 1.03 0.00 0.00 0.00 0.00 0.00 0.00 0.00 0.00 0.37 2.32 1.98 3.43

Grammatophora oceanica 0.00 0.00 0.00 0.00 0.00 0.00 0.52 0.00 0.00 0.54 0.00 1.16 0.86 0.69 0.39 0.37 0.00 0.00 0.26

Gyrosigma balticum 0.00 0.62 2.05 2.47 2.69 3.48 8.25 3.95 5.87 4.04 1.57 0.87 2.31 3.99 1.75 1.11 0.00 0.56 0.79

Luticula mutica 1.67 0.31 2.37 1.32 6.85 3.77 4.64 2.96 4.11 3.23 0.94 2.03 3.17 3.65 4.09 0.93 1.66 0.56 1.85

Mastogloia lanceolata 0.00 0.00 1.10 0.33 0.73 1.45 0.77 1.32 1.17 0.27 0.00 0.00 0.58 0.00 0.00 0.93 0.00 0.00 0.00

Mastogloia pusilla 0.00 0.62 5.68 0.49 0.73 2.03 1.29 2.30 1.17 1.35 0.63 1.16 0.86 0.87 0.00 0.00 0.00 0.00 0.00

Navicula cryptocephala 0.00 1.23 5.68 0.82 1.96 3.48 2.58 2.96 3.23 0.81 0.63 1.45 0.86 0.52 1.17 0.93 0.00 0.00 0.00

Navicula digitoradiata 0.00 0.00 0.00 0.00 0.00 0.00 0.77 0.00 1.47 2.16 1.25 1.45 2.02 1.91 1.36 2.04 0.00 0.00 0.00

Navicula halophila 2.15 0.31 1.42 4.11 2.93 6.09 5.93 6.91 3.52 1.35 0.63 1.45 1.15 0.87 0.58 0.93 0.00 0.00 0.00

Navicula incertata 0.00 0.62 6.62 34.54 5.38 1.16 0.77 0.66 0.00 0.00 0.00 0.00 0.00 0.00 1.17 4.26 2.32 4.24 4.22

Navicula lanceolate 0.00 0.00 0.00 0.00 0.00 0.00 0.00 0.00 0.00 0.00 0.00 0.00 0.00 0.00 0.00 0.00 1.32 0.28 1.85

Navicula lyra 0.00 0.00 0.00 0.00 0.00 0.00 0.00 0.00 0.00 0.27 2.51 0.87 2.02 0.69 0.00 0.00 0.00 0.00 0.00

Navicula tripunctata 2.15 2.47 11.20 3.12 5.87 12.17 5.67 10.86 6.74 1.08 4.70 5.51 6.34 3.82 0.78 0.74 0.66 0.56 2.37

Nitzschia longissima 0.00 0.00 0.00 0.00 0.00 0.00 0.00 0.00 0.59 5.66 17.24 6.38 1.73 1.39 0.39 0.00 0.00 0.00 0.00

Nitzschia obtusa 0.00 0.00 0.00 0.00 0.00 0.58 2.32 3.62 4.40 5.66 3.45 6.09 5.19 9.55 4. 29 2.78 0.00 0.85 2.90

Nitzschia palustris 6.22 1.85 1.42 0.33 0.00 0.00 0.00 0.00 0.00 0.00 0.00 0.00 0.00 0.00 3.12 0.56 1.66 3.10 5.80

Nitzschia pusilla 5.98 2.47 0.95 1.15 0.73 0.58 0.52 0.33 1.17 0.54 2.51 2.61 3.17 1.04 6.24 13.89 18.87 15.25 10.29

Nitzschia sigma 0.00 0.00 20.19 3.62 5.13 6.96 3.87 8.22 5.28 2.16 3.76 2.61 1.44 4.51 0.78 0.37 0.00 0.56 1.06

Nitzschia sinuata 0.00 0.00 0.47 0.16 0.00 0.00 0.00 0.00 0.00 0.00 0.00 0.00 0.00 0.00 0.00 0.00 0.00 0.00 0.00

Nitzschia tryblionella 0.48 2.78 0.00 0.00 0.00 0.00 0.00 0.00 0.00 0.00 0.94 0.00 0.00 0.69 3.31 1.11 0.66 3.11 3.17

Paralia sulcata 0.24 0.31 0.47 0.33 0.49 1.45 0.52 1.32 1.17 1.62 0.63 0.87 2.31 0.35 1.56 1.30 0.00 0.00 0.00

Pinnularia subcapitata 2.63 0.31 0.00 0.16 0.00 0.00 0.00 0.00 0.00 0.00 0.00 0.00 0.00 0.00 0.00 0.00 3.64 0.85 1.85

Plagiogramma vanheurckii 0.24 0.62 0.00 0.00 0.00 0.29 0.52 0.66 1.17 0.81 0.31 0.58 0.58 0.52 0.00 0.00 0.00 0.00 0.00

Rhopalodia gibberula 0.00 0.31 3.94 0.99 1.71 3.48 1.80 5.92 2.05 2.16 0.94 0.58 0.58 0.35 0.39 0.93 0.00 0.00 0.00

Synedra acus 2.63 1.85 1.58 0.99 0.73 0.87 0.52 0.00 0.00 0.00 0.00 0.00 0.00 0.00 0.00 0.00 2.32 2.82 2.37

Synedra tabulata 0.00 0.00 0.32 0.33 0.98 1.74 3.87 3.29 3.52 1.35 10.34 6.67 1.73 2.26 1.75 1.30 0.00 0.85 0.00

Thalassiosira nitzschioides 0.00 0.00 0.00 0.00 0.00 0.00 0.26 0.00 0.59 3.23 1.88 2.90 3.46 5.38 4.87 1.85 0.00 0.28 1.06

**Marine water 0.48 1.54 2.84 0.82 2.44 4.64 6.70 5.59 6.74 10.24 8.78 12.75 14.99 15.97 9.55 7.22 0.99 2.54 2.11**

**Brackish water 19.86 20.99 69.24 85.36 71.64 71.01 65.46 74.34 73.90 78.44 77.43 72.17 66.57 68.92 41.33 40.19 6.95 14.97 14.51**

**Fresh to brackish water 4.31 6.79 19.72 7.07 20.05 22.03 18.04 18.42 16.42 8.36 10.97 11.59 12.68 11.81 7.02 5.93 4.30 4.80 6.86**

**Freshwater 72.73 70.37 8.20 6.58 5.87 2.32 9.79 1.64 2.93 2.96 2.82 3.48 5.76 3.30 42.11 46.67 84.11 76.84 74.67**

**Freshwater (no salt) 2.63 0.31 0.00 0.16 0.00 0.00 0.00 0.00 0.00 0.00 0.00 0.00 0.00 0.00 0.00 0.00 3.64 0.85 1.85**
