# Supplementary material for: Diatoms and pollen data from modern surface sediment samples collected from the Merang wetlands, Kuala Terengganu, Malaysia
Source: Data Brief. 2018 Nov 3;21:1886–9. doi: 10.1016/j.dib.2018.10.156 (PMC6260407; doi:10.1016/j.dib.2018.10.156)
Supplement: Supplementary file 3 — Supplementary material [file mmc3.docx]

Percentages of total land pollen from each modern surface sediment sample collected from the Merang wetlands, Kuala Terengganu, Malaysia

**Site SI5 SI4 SG1 SG2 SG3 SH1 SF2 SE4 SF4 SF6 SF5 SM13 SF3 SM11 SE3 SE2 SM12**

**Elevation (m, MSL) +1.45 +1.25 +0.95 +0.83 +0.72 +0.65 +0.54 +0.48 +0.46 +0.42 +0.41 +0.40 +0.40 +0.38 +0.35 +0.31 +0.24**

Bruguiera/Ceriops 1.93 2.65 2.50 0.36 0.00 0.29 18.43 8.36 27.50 9.47 15.07 0.92 18.52 1.28 6.88 7.42 4.07

Rhizophora 1.93 1.99 7.50 7.66 0.79 0.58 32.08 43.27 20.00 20.35 20.89 30.28 25.93 18.80 48.91 35.69 24.81

Sonnaratia 0.00 0.00 0.00 0.00 0.00 0.00 0.00 0.00 0.00 0.00 0.00 0.00 0.00 0.00 0.00 0.00 0.00

Acrostichum 0.32 0.00 0.71 3.65 1.57 0.00 2.73 0.36 0.36 0.00 3.08 0.46 0.00 1.71 0.00 0.35 0.00

Brownlowia 0.32 0.00 0.00 0.00 0.00 0.00 0.00 0.00 0.00 0.00 0.00 0.46 0.34 0.00 0.36 0.00 0.00

Excoecaria agallocha 0.32 1.66 0.36 1.09 0.39 0.00 18.43 2.91 12.50 23.51 25.68 2.75 10.77 11.11 3.26 1.41 1.85

Heritiera 0.00 0.00 0.00 0.00 0.39 0.00 0.00 0.00 0.00 0.00 0.00 0.00 0.00 0.00 0.00 0.00 0.00

Lumnitzera 0.00 0.00 0.00 0.00 0.00 0.00 0.34 0.00 0.36 0.00 0.00 0.46 1.68 0.00 1.45 0.35 0.00

Oncosperma 0.32 0.99 2.86 2.92 2.76 0.58 5.12 2.91 6.07 5.26 8.22 0.46 6.73 0.85 2.90 5.30 0.00

Xylocarpus 0.00 0.33 3.57 0.00 0.00 0.58 1.37 1.82 0.71 1.05 1.37 11.01 1.35 23.93 3.99 0.35 20.37

Barringtonia 0.00 0.00 1.43 1.09 0.79 0.00 0.34 0.36 0.00 0.00 0.00 0.00 0.67 0.00 0.36 0.00 0.00

Campnosperma 0.00 0.00 7.50 4.38 1.18 0.29 0.00 1.45 0.00 0.35 0.00 0.00 1.01 0.43 1.45 0.71 1.85

Castanopsis/Lithocarpus 0.64 0.33 1.07 0.36 0.39 0.29 0.00 0.00 0.00 0.00 0.00 0.92 0.34 1.71 0.36 0.71 0.74

Casuarina equisetifolia 1.93 38.41 0.71 2.55 0.79 0.00 1.71 1.45 1.79 8.07 6.16 10.09 1.01 4.70 4.35 3.53 2.59

Dipterocarpaceae 0.00 0.00 6.43 9.12 38.19 0.00 0.68 0.36 0.71 0.00 0.00 1.83 0.00 1.71 1.09 1.41 4.44

Gluta-type 0.64 0.00 1.79 0.00 0.00 0.00 0.00 0.36 0.00 0.00 0.00 0.00 0.00 0.00 0.36 0.00 0.00

Ilex 0.32 1.66 4.29 8.76 8.27 0.00 0.34 3.27 1.79 2.11 0.68 0.46 3.37 0.85 0.72 4.24 2.96

Mallotus 0.32 0.00 0.00 0.00 1.57 0.00 0.34 0.36 0.00 0.00 0.00 0.00 0.34 0.00 0.36 0.00 0.00

Melaleuca cajuputi (Gelam) 0.96 1.32 0.71 8.39 2.36 27.54 2.05 1.09 6.07 2.81 0.68 11.93 5.39 5.98 0.72 6.01 5.56

Mimosaceae 0.64 0.00 0.00 0.00 0.00 0.00 0.00 0.73 0.00 0.00 0.00 0.00 0.00 0.00 1.45 0.00 0.00

Myrica 0.32 0.66 1.79 25.55 14.96 0.87 0.68 1.82 1.79 0.35 1.03 0.92 0.67 0.85 3.26 3.18 7.04

Myrtaceae 72.03 44.70 18.57 0.00 0.00 56.52 4.10 15.27 12.86 7.02 6.51 5.50 10.77 5.13 5.80 10.95 5.56

Nephelium 0.00 0.00 0.36 0.36 0.00 0.00 0.00 0.00 0.00 0.00 0.00 0.00 0.34 0.00 0.00 0.00 0.00

Nuphar 0.00 0.00 0.00 0.00 0.00 0.00 0.00 0.00 0.00 0.00 0.00 0.00 0.00 0.00 0.00 0.71 0.00

Stemonurus 0.00 0.00 0.00 0.00 0.00 0.00 0.34 0.36 0.36 0.70 0.00 0.00 0.00 0.00 0.36 1.41 0.00

Utricularia 0.00 0.00 0.00 0.00 0.00 0.00 0.00 0.00 0.00 0.00 0.00 0.00 0.00 0.00 0.00 0.00 0.00

Celtis 0.00 0.00 0.36 0.00 0.39 0.00 0.68 0.00 0.00 0.00 0.34 0.00 0.00 0.00 0.72 0.35 0.00

Chenopodiaceae 0.00 0.33 0.36 0.36 0.00 0.00 0.00 0.00 0.00 0.00 0.00 0.00 0.00 0.00 0.00 0.00 0.00

Conocarpus 0.00 0.00 0.00 0.00 0.00 0.00 0.00 0.36 0.00 0.00 0.00 0.00 0.00 0.00 0.00 0.00 0.00

Macaranga 0.00 0.00 10.00 4.01 0.79 0.00 0.00 0.00 0.00 0.00 0.00 0.00 0.00 0.00 0.72 0.00 0.00

Poaceae 1.93 0.99 4.29 12.04 7.09 8.12 2.73 4.00 1.43 4.91 0.68 3.67 1.68 3.85 1.45 2.12 2.59

Terminalia 0.00 0.00 0.00 0.00 0.00 0.00 0.00 0.00 0.00 0.00 0.00 0.00 0.00 0.00 0.00 0.00 0.00

Trema 1.29 0.33 0.36 0.00 0.00 0.29 0.68 1.09 0.71 0.00 0.34 0.00 0.00 0.00 0.72 1.06 0.00

Utricaceae 0.00 0.00 0.36 0.73 0.00 0.00 0.00 0.36 0.00 0.00 0.00 0.00 0.00 0.00 0.00 0.00 0.00

Cyperaceae 11.58 1.32 3.57 1.46 3.15 2.32 4.78 1.82 3.21 9.47 2.40 3.67 5.05 0.85 0.72 7.42 1.48

Nypa frusticans 0.64 0.33 0.00 0.00 0.00 0.29 0.34 0.00 0.00 1.05 0.68 11.01 0.67 12.82 0.00 0.00 10.37

Pandanus 0.00 0.00 9.29 0.00 0.00 0.29 0.34 1.82 1.79 0.35 0.34 0.00 0.67 0.00 2.17 2.83 0.00

Polygonum 0.00 0.00 0.00 1.09 0.00 0.00 0.00 0.00 0.00 0.00 0.00 0.00 0.00 0.00 0.00 0.00 0.00

Aglaia 0.00 0.00 0.00 0.00 0.00 0.00 0.00 0.73 0.00 0.00 0.00 0.00 0.00 0.00 0.36 0.00 0.00

Aidia 0.00 0.00 1.07 0.36 0.00 0.00 0.00 0.00 0.00 0.00 0.00 0.00 0.00 0.00 0.00 0.00 0.00

Altingia 0.00 0.00 0.00 0.00 0.00 0.00 0.00 0.00 0.00 0.00 0.00 0.00 0.00 0.00 0.00 0.00 0.00

Anacardiaceae 0.00 0.00 0.00 0.00 0.00 0.00 0.00 0.00 0.00 0.00 0.00 0.00 0.00 0.00 0.00 0.00 0.00

Araliaceae 0.00 0.00 0.00 0.00 0.00 0.00 0.00 0.00 0.00 0.00 0.00 0.00 0.00 0.00 0.00 0.00 0.00

Betula 0.00 0.00 0.00 0.00 0.39 0.00 0.34 0.00 0.00 0.00 0.00 0.00 0.00 0.00 0.36 0.00 0.00

Chrysophyllum 0.00 0.00 0.00 0.73 0.79 0.00 0.00 0.36 0.00 0.00 0.00 0.00 0.00 0.00 0.00 0.00 0.00

Combretaceae 0.00 0.00 0.71 0.00 0.00 0.00 0.00 0.00 0.00 0.00 0.00 0.00 0.00 0.00 0.00 0.00 0.00

Elaeocarpus 0.00 0.00 2.14 0.00 0.00 0.87 0.00 0.36 0.00 0.00 0.34 0.00 0.00 0.00 0.72 0.00 0.00

Euphora 0.00 0.00 0.00 0.00 0.00 0.00 0.00 0.00 0.00 0.00 0.00 0.00 0.00 0.00 0.00 0.00 0.00

Euphorbiaceae 0.32 1.66 0.71 0.73 0.39 0.00 0.34 0.00 0.00 1.75 4.79 0.46 1.68 0.85 0.72 1.06 3.70

Ficus 0.00 0.00 0.36 0.00 0.00 0.00 0.34 0.36 0.00 0.00 0.00 0.00 0.00 0.00 0.00 0.35 0.00

Ixora 0.00 0.00 0.00 0.73 0.39 0.00 0.00 0.36 0.00 0.00 0.00 0.00 0.00 0.00 0.00 0.00 0.00

Labiatae 0.00 0.00 0.00 0.00 0.39 0.00 0.00 0.00 0.00 0.00 0.00 0.00 0.00 0.00 0.00 0.00 0.00

Loranthaceae 0.00 0.00 0.71 0.00 0.39 0.00 0.00 0.00 0.00 0.00 0.00 0.00 0.00 0.00 0.00 0.00 0.00

Melastoma 0.00 0.00 0.00 0.00 0.00 0.00 0.00 0.00 0.00 0.00 0.00 1.83 0.67 0.43 0.00 0.00 0.00

Melastomataceae 0.32 0.00 0.00 0.00 0.00 0.29 0.00 0.73 0.00 0.70 0.00 0.00 0.00 0.00 0.36 0.00 0.00

Memecylon 0.00 0.00 0.00 0.00 0.00 0.00 0.00 0.00 0.00 0.00 0.00 0.00 0.00 0.00 0.00 0.71 0.00

Pilea 0.00 0.00 0.00 0.00 0.00 0.00 0.00 0.00 0.00 0.00 0.00 0.00 0.00 0.00 0.00 0.00 0.00

Pinus 0.00 0.33 0.36 0.00 0.00 0.00 0.00 0.00 0.00 0.70 0.68 0.00 0.00 0.00 0.00 0.00 0.00

Pometia 0.00 0.00 0.00 0.00 0.00 0.00 0.00 0.00 0.00 0.00 0.00 0.00 0.00 0.00 0.00 0.00 0.00

Rubiaceae 0.00 0.00 0.71 0.00 0.00 0.00 0.00 0.00 0.00 0.00 0.00 0.00 0.00 1.28 0.36 0.00 0.00

Rutaceae 0.00 0.00 0.00 0.00 0.00 0.00 0.00 0.00 0.00 0.00 0.00 0.00 0.00 0.00 0.00 0.00 0.00

Sapindaceae 0.64 0.00 2.50 0.00 0.00 0.00 0.00 0.73 0.00 0.00 0.00 0.92 0.34 0.00 1.45 0.00 0.00

Sapindus 0.00 0.00 0.00 0.00 0.00 0.00 0.34 0.00 0.00 0.00 0.00 0.00 0.00 0.00 0.00 0.00 0.00

Sapium 0.00 0.00 0.00 0.00 0.39 0.00 0.00 0.00 0.00 0.00 0.00 0.00 0.00 0.00 0.00 0.00 0.00

Sterculiaceae 0.00 0.00 0.00 0.00 0.00 0.00 0.00 0.00 0.00 0.00 0.00 0.00 0.00 0.00 0.00 0.00 0.00

Toona 0.00 0.00 0.00 0.00 0.00 0.00 0.00 0.00 0.00 0.00 0.00 0.00 0.00 0.00 0.00 0.35 0.00

Toxicodendron 0.32 0.00 0.00 0.36 9.06 0.00 0.00 0.36 0.00 0.00 0.00 0.00 0.00 0.00 0.36 0.00 0.00

Ulmaceae 0.00 0.00 0.00 0.00 0.00 0.00 0.00 0.00 0.00 0.00 0.00 0.00 0.00 0.00 0.00 0.00 0.00

Ulmus 0.00 0.00 0.00 1.09 1.97 0.00 0.00 0.00 0.00 0.00 0.00 0.00 0.00 0.00 0.36 0.00 0.00

**Mangrove 3.86 4.64 10.00 8.03 0.79 0.87 50.51 51.64 47.50 29.82 35.96 31.19 44.44 20.09 55.80 43.11 28.89**

**Back-Mangrove 1.93 3.31 7.50 8.76 5.12 1.45 28.33 8.00 20.00 30.88 39.04 26.61 21.55 50.43 11.96 7.77 32.59**

**Gelam 77.81 87.09 63.93 64.60 69.29 85.80 10.92 28.73 27.14 21.75 15.41 31.65 24.58 21.37 23.55 35.69 30.74**

**Open ground vegetation 14.79 2.98 9.29 14.60 10.63 10.72 8.87 7.64 5.36 14.39 3.77 7.34 6.73 4.70 3.62 10.95 4.07**

**Swamp 0.00 0.00 0.00 0.00 0.00 0.00 0.00 0.00 0.00 0.00 0.00 0.00 0.00 0.00 0.00 0.00 0.00**

**Upland forest 1.61 1.99 9.29 4.01 14.17 1.16 1.37 4.00 0.00 3.16 5.82 3.21 2.69 3.42 5.07 2.47 3.70**

**Site SE1 SK1 SM09 SM03 SM14 SM15 SM16 SH2 SH3 SL3 SL2 SL1**

**Elevation (m, MSL) +0.22 +0.18 +0.17 +0.14 -0.01 -0.09 -0.15 +0.09 -0.41 +0.19 -0.23 -1.19**

Bruguiera/Ceriops 16.37 21.37 1.27 3.92 13.97 9.22 4.20 2.23 8.23 1.57 1.00 0.00

Rhizophora 29.18 43.59 19.83 23.53 17.88 19.35 24.48 14.53 9.49 5.51 15.00 22.40

Sonnaratia 0.00 0.00 0.00 0.00 0.00 0.00 0.00 0.56 0.00 2.36 1.00 2.40

Acrostichum 0.36 0.85 1.27 3.14 1.68 4.61 10.49 0.00 0.00 1.57 2.00 0.80

Brownlowia 0.00 0.00 0.00 0.00 0.00 0.00 0.00 0.00 0.00 0.00 0.00 0.00

Excoecaria agallocha 2.14 2.99 6.33 8.63 3.91 4.15 8.74 5.59 0.00 0.00 1.00 1.60

Heritiera 0.00 0.00 0.00 0.00 0.00 0.00 0.00 0.00 0.00 0.00 0.00 0.00

Lumnitzera 1.07 0.43 0.00 0.00 0.00 0.00 0.00 0.00 0.00 0.00 0.00 0.00

Oncosperma 3.91 0.43 1.27 5.49 3.91 2.30 0.35 6.70 2.53 3.15 4.00 0.00

Xylocarpus 3.20 0.43 27.43 16.86 17.32 30.41 22.03 5.59 3.80 4.72 4.00 5.60

Barringtonia 0.36 0.00 0.00 0.00 0.00 0.00 0.00 0.00 0.00 0.00 0.00 0.00

Campnosperma 3.20 0.43 0.84 0.78 0.56 1.84 2.45 3.91 3.16 9.45 4.00 6.40

Castanopsis/Lithocarpus 2.14 0.85 2.95 4.31 2.23 0.92 0.35 1.12 3.80 1.57 0.00 1.60

Casuarina equisetifolia 2.49 7.26 3.38 1.18 4.47 1.84 1.40 0.00 1.90 2.36 0.00 0.00

Dipterocarpaceae 0.36 0.00 1.69 0.39 1.12 0.46 0.35 0.00 1.90 0.00 4.00 0.80

Gluta-type 1.42 0.00 0.00 0.00 0.00 0.00 0.00 0.00 0.00 0.00 0.00 0.00

Ilex 2.49 0.43 1.27 0.39 0.00 0.92 1.75 6.15 1.27 3.94 0.00 1.60

Mallotus 0.71 1.28 0.00 0.00 0.00 0.00 0.00 0.00 0.00 0.00 0.00 0.00

Melaleuca cajuputi (Gelam) 3.91 2.56 7.17 2.75 2.23 0.92 1.75 0.00 0.00 0.00 0.00 0.00

Mimosaceae 0.00 0.00 0.00 0.00 0.00 0.00 1.05 0.00 0.00 0.00 5.00 0.80

Myrica 5.34 0.43 0.84 1.96 0.56 0.92 0.35 0.00 0.00 0.00 0.00 0.00

Myrtaceae 7.12 5.13 2.95 5.49 17.88 2.30 3.85 19.55 47.47 14.96 23.00 33.60

Nephelium 0.00 0.00 0.00 0.00 0.00 0.00 0.00 0.00 0.00 0.00 0.00 0.00

Nuphar 0.00 0.00 0.00 0.00 0.00 0.00 0.00 0.00 0.00 0.00 0.00 0.00

Stemonurus 1.78 0.43 0.00 0.00 0.00 0.00 0.00 0.00 0.00 0.00 0.00 0.00

Utricularia 0.00 0.00 0.00 0.00 0.00 0.00 0.00 0.00 0.00 0.00 0.00 0.00

Celtis 0.36 0.00 0.00 0.00 0.00 0.00 0.00 0.00 0.00 0.00 0.00 0.00

Chenopodiaceae 0.36 0.00 0.00 0.00 0.00 0.00 0.00 0.00 0.00 0.00 0.00 0.00

Conocarpus 0.00 0.00 0.00 0.00 0.00 0.00 0.00 0.00 0.00 0.00 0.00 0.00

Macaranga 0.00 0.00 0.00 0.00 0.00 0.00 0.00 0.00 0.00 0.00 0.00 0.00

Poaceae 2.14 1.28 2.11 10.20 6.15 8.76 8.74 12.85 1.27 7.87 7.00 6.40

Terminalia 0.00 0.00 0.00 0.00 0.00 0.00 0.00 0.00 0.00 0.00 0.00 0.00

Trema 1.78 2.99 0.00 0.00 0.00 0.00 0.00 0.00 0.00 0.00 0.00 0.00

Utricaceae 0.36 0.00 0.00 0.00 0.00 0.00 0.00 0.00 0.00 0.00 0.00 0.00

Cyperaceae 4.27 0.43 1.69 0.78 0.00 5.07 4.20 2.23 3.16 25.20 6.00 4.80

Nypa frusticans 0.00 1.28 10.55 8.24 3.91 4.15 1.75 2.23 1.27 2.36 6.00 1.60

Pandanus 0.00 0.00 0.00 0.00 0.00 0.00 1.05 1.12 0.00 0.79 0.00 0.00

Polygonum 0.00 0.00 0.00 0.00 0.00 0.00 0.00 0.00 0.00 0.00 0.00 0.00

Aglaia 1.07 0.43 0.00 0.00 0.00 0.00 0.00 0.00 0.63 0.00 0.00 0.00

Aidia 0.36 0.00 0.00 0.00 0.00 0.00 0.00 0.00 0.00 0.00 0.00 0.00

Altingia 0.00 0.00 0.00 0.00 0.00 0.00 0.00 0.00 0.00 0.00 0.00 0.00

Anacardiaceae 0.00 0.00 0.00 0.00 0.00 0.00 0.00 0.00 0.00 0.00 0.00 0.00

Araliaceae 0.00 0.00 1.69 0.00 1.12 0.00 0.00 1.68 4.43 4.72 4.00 2.60

Betula 0.00 0.00 0.00 0.00 0.00 0.00 0.00 0.00 0.00 0.00 0.00 0.00

Chrysophyllum 0.00 0.00 0.00 0.00 0.00 0.00 0.00 0.00 0.00 0.00 0.00 0.00

Combretaceae 0.36 0.43 0.00 0.00 0.00 0.00 0.00 0.00 0.00 0.00 0.00 0.00

Elaeocarpus 0.00 0.00 0.00 0.00 0.00 0.00 0.00 1.12 1.90 1.57 2.00 2.40

Euphora 0.00 0.00 0.00 0.00 0.00 0.00 0.00 0.00 0.00 0.00 0.00 0.00

Euphorbiaceae 0.00 1.28 0.00 0.00 0.00 0.00 0.00 0.00 0.00 0.00 0.00 0.00

Ficus 0.00 0.43 0.00 0.00 0.00 0.00 0.00 0.00 0.00 0.00 0.00 0.00

Ixora 0.00 0.00 0.00 0.00 0.00 0.00 0.00 0.00 0.00 0.00 0.00 0.00

Labiatae 0.00 0.00 0.00 0.00 0.00 0.00 0.00 0.00 0.00 0.00 0.00 0.00

Loranthaceae 0.00 0.00 0.00 0.00 0.00 0.00 0.00 0.00 0.00 0.00 0.00 0.00

Melastoma 0.00 0.00 4.64 1.96 1.12 1.84 0.70 7.82 3.80 4.72 7.00 4.80

Melastomataceae 0.00 0.85 0.00 0.00 0.00 0.00 0.00 0.00 0.00 0.00 0.00 0.00

Memecylon 0.36 0.00 0.00 0.00 0.00 0.00 0.00 0.00 0.00 0.00 0.00 0.00

Pilea 0.00 0.00 0.00 0.00 0.00 0.00 0.00 0.00 0.00 0.00 0.00 0.00

Pinus 0.36 0.43 0.00 0.00 0.00 0.00 0.00 0.00 0.00 0.00 0.00 0.00

Pometia 0.00 0.00 0.00 0.00 0.00 0.00 0.00 0.00 0.00 0.00 0.00 0.00

Rubiaceae 0.00 0.00 0.00 0.00 0.00 0.00 0.00 0.00 0.00 0.79 0.00 0.00

Rutaceae 0.00 0.00 0.00 0.00 0.00 0.00 0.00 0.00 0.00 0.00 0.00 0.00

Sapindaceae 0.00 0.43 0.00 0.00 0.00 0.00 0.00 3.91 0.00 0.00 2.00 0.00

Sapindus 0.00 0.43 0.00 0.00 0.00 0.00 0.00 0.00 0.00 0.00 0.00 0.00

Sapium 0.00 0.00 0.00 0.00 0.00 0.00 0.00 0.00 0.00 0.00 0.00 0.00

Sterculiaceae 0.00 0.00 0.00 0.00 0.00 0.00 0.00 1.12 0.00 0.79 2.00 0.00

Toona 0.00 0.00 0.84 0.00 0.00 0.00 0.00 0.00 0.00 0.00 0.00 0.00

Toxicodendron 0.00 0.00 0.00 0.00 0.00 0.00 0.00 0.00 0.00 0.00 0.00 0.00

Ulmaceae 0.00 0.00 0.00 0.00 0.00 0.00 0.00 0.00 0.00 0.00 0.00 0.00

Ulmus 0.71 0.43 0.00 0.00 0.00 0.00 0.00 0.00 0.00 0.00 0.00 0.00

**Mangrove 45.55 64.96 21.10 27.45 31.84 28.57 28.67 17.32 17.72 9.45 17.00 24.80**

**Back-Mangrove 10.68 6.41 46.84 42.35 30.73 45.62 43.36 20.11 7.59 11.81 17.00 9.60**

**Gelam 31.32 18.80 21.10 17.25 29.05 10.14 14.34 31.48 59.49 33.07 36.00 44.80**

**Open ground vegetation 9.25 4.70 3.80 10.98 6.15 13.82 12.94 12.85 1.27 7.87 7.00 6.40**

**Swamp 0.00 0.00 0.00 0.00 0.00 0.00 0.00 2.23 3.16 25.20 6.00 4.80**

**Upland forest 3.20 5.13 7.17 1.96 2.23 1.84 0.70 15.64 10.76 12.60 17.00 9.60**
